# Multivariate Whole Brain‐Cognitive‐Clinical Severity Mapping in Alzheimer's Disease Continuum using Explainable AI

**DOI:** 10.1002/alz70856_101044

**Published:** 2025-12-24

**Authors:** Taslim Murad, Hui‐Yuan Miao, Deepa S. Thakuri, Gauri Darekar, Ganesh Chand

**Affiliations:** ^1^ Washington University in St. Louis, St. Louis, MO, USA; ^2^ University of Missouri, Columbia, MO, USA

## Abstract

**Background:**

Brain volumetric changes quantified by MRI are associated with cognitive declines in Alzheimer's disease (AD). Prior univariate approaches have established correlations for some individual brain‐behavior measures, but the whole brain (WB)‐cognition multivariate predictive relationships are yet to be systematically investigated.

**Method:**

The integration of advanced explainable artificial intelligence (AI) techniques with WB volumetric changes has significant potential to capture complex multivariate WB‐cognition relationships in the AD continuum. Herein we utilized various machine learning (ML) models along with a deep learning (DL) model to perform cognition prediction in the AD continuum based on the WB regional features extracted from MRI data. The global cognition was assessed through the Mini‐Mental State Examination (MMSE). Moreover, the optimal predictive DL model was integrated with the Shapley Additive exPlanations (SHAP) feature importance strategy, referred to as DL‐SHAP, to identify the hierarchy of multivariate significant brain regions involved in cognitive prediction in the AD continuum. The DL‐SHAP model was initially validated on semi‐simulated data (*n* = 1108) and then applied to the actual experimental data (*n* = 668; age: 55.1‐91.5 years; 46.1% females). Finally, the severity of AD characteristics, measured by the Clinical Dementia Rating‐Sum of Boxes (CDR‐SB), was assessed within the framework of DL‐SHAP with the goal of identifying the key brain regional metrics contributing to disease severity processes.

**Result:**

The DL model tremendously outperformed the conventional ML models for MMSE prediction using the MRI‐based WB volumetric changes data. The DL‐SHAP model portrayed robust performance on semi‐simulated data by achieving a Spearman's correlation of 0.94 between the actual and predicted MMSE scores as well as capturing dominant perturbed brain regions. It also yielded excellent performance on the experimental data by demonstrating a Spearman's correlation of 0.96 and identified several hierarchically dominant brain regions associated with MMSE estimation in the AD continuum. Additionally, DL‐SHAP captured various key brain regions involved in AD severity.

**Conclusion:**

The sophisticated explainable AI method, DL‐SHAP, showed robust performance in predicting the global cognition using a large MRI dataset, along with identifying the multivariate WB‐cognition relationships. Additionally, it portrayed compelling evidence for predicting clinical severity and identified the dominant brain regions that significantly contributed to these predictions.

